# Topological control of nitric oxide secretion by tantalum oxide nanodot arrays

**DOI:** 10.1186/s12951-015-0144-y

**Published:** 2015-11-09

**Authors:** Udesh Dhawan, Chia Hui Lee, Chun-Chung Huang, Ying Hao Chu, Guewha S. Huang, Yan-Ren Lin, Wen-Liang Chen

**Affiliations:** Department Material Science and Technology, National Chiao Tung University Hsinchu, 1001 University Road, Hsinchu, 300 Taiwan, ROC; Hokan Life Technology, F2, 793 Fu-Ke Road, Taichung, Taiwan, ROC; Department of Emergency Medicine, Changhua Christian Hospital, 135 Nanshiao Street, Changhua, 500 Taiwan; Department of Biological Science and Technology, National Chiao Tung University Hsinchu, 1001 University Road, Hsinchu, 300 Taiwan, ROC

**Keywords:** Tantalum oxide, Nanodots, Cardiomyocytes, eNOS, iNOS, Signaling pathway

## Abstract

**Background:**

Nitric oxide (NO) plays a very important role in the cardiovascular system as a major secondary messenger in signaling pathway. Its concentration regulates most of the important physiological indexes including the systemic blood pressure, blood flow, regional vascular tone and other cardiac functions. The effect of nanotopography on the NO secretion in cardiomyocytes has not been elucidated before. In this study, we report how the nanotopography can modulate the secretion profile of NO and attempt to elucidate the genetic pathways responsible for the same by using Tantalum Oxide nanodot arrays ranging from 10 to 200 nm. A series of nanodot arrays were fabricated with dot diameter ranging from 10 to 200 nm. Temporal NO release of cardiomyocytes was quantified when grown on different surfaces. Quantitative RT-PCR and Western blot were performed to verify the genetic pathways of NO release.

**Results:**

After hours 24 of cell seeding, NO release was slowly enhanced by the increase of dot diameter from 10 nm up to 50 nm, mildly enhanced to a medium level at 100 nm, and increase rapidly to a high level at 200 nm. The temporal enhancement of NO release dropped dramatically on day 3. On day 5, a topology-dependent profile was established that maximized at 50 nm and dropped to control level at 200 nm. The NO releasing profile was closely associated with the expression patterns of genes associated with Endothelial nitric oxide synthase (eNOS) pathway [GPCR, PI3K, Akt, Bad, Bcl-2, NFκB(p65), eNOS], but less associated with Inducible nitric oxide synthase (iNOS) pathway (TNF-α, ILK, Akt, IκBα, NFκB, iNOS). Western blotting of Akt, eNOS, iNOS, and NFκB further validated that eNOS pathway was modulated by nanotopology.

**Conclusions:**

Based on the findings of the present study, 50, 100 nm can serve as the suitable nanotopography patterns for cardiac implant surface design. These two nanodot arrays promote NO secretion and can also promote the vascular smooth muscle relaxation. The results of this study can improve the heart stent design in the medical treatments.

## Background

Nanotopography affects cell physiology. But the effects of different size of nanosurfaces on different cells are very inconsistent. Topographies like nanodots [1–4] nano-islands [5], nano-concave [6], nanocrystalline [7] diamond, nano-groove [8–11] nanotube [12], nano-ridge [13, 14], nanopore [15] have been reported to affect the cell physiological behavior, including biocompatibility, cell growth, migration and cell adhesion. Previous studies conducted on the interaction of the cells with quartz surfaces have shown change in the surface area of the cells [16]. Change in the morphologies of osteoblasts on interaction with substrates has also been shown [17, 18]. Roughness of Titanium substrates has also been seen to modulate the adhesions of the cells to the nanosurface [19]. We have shown in our previous studies that cells have maximum adhesion area on 50 nm nanodots whereas 100 and 200 nm always trigger off immune response [20] after day 3. For nanoislands, the cytoskeletal organization, cell growth and proliferation are highly enhanced on size smaller than 27 nm but decreased cell physiological indexes are observed for size greater than 80 nm [21]. Previous studies have shown that C3A cells spread and grew confluent with elongated and aligned morphology along 100 nm nanogrooves [22] Similar studies have also been conducted on Titanium dioxide nanotubes (TiO_2_) for 15 nm spaced incubation but deteriorated cell proliferation has been observed on 350 nm nanosurface [15].

We have reported in our previous studies on NIH-3T3 cells that nanodot arrays with 10–200 nm nanodots modulate cell adhesion and induce an apoptosis like abnormality [2]. The apoptosis like abnormality became evident in 200 nm nanodots after day 1. The abnormality starts to show on 50 nm after day 3. 40 and 75 nm nanopores promoted cell adhesion and migration in fibroblast by controlling expression of integrins and ERK1/2 in a time dependent manner [4]. A lot of variability in the cell morphology, migration ability, gene and protein expression was found after 12, 24, 48, hours 72 and became indistinguishable after incubation for hours 120.

Previous studies conducted on cardiomyocytes have shown maximum growth, proliferation and extended morphology on 50 nm nanodot arrays after day 3. Maximum growth and adhesion shifted to 100 nm after incubation for hours 120 [3]. This shows that nanotopography regulates cell physiology not only in the size dependent manner but also in a time dependent manner.

Nitric oxide (NO) plays a very important role in the cardiovascular system as a major secondary messenger in signaling pathway. It is a key signaling messenger in the cardiovascular system. Its concentration regulates most of the important physiological indexes including the systemic blood pressure, blood flow, regional vascular tone and other cardiac functions. It also maintains the vascular integrity by inhibiting the platelet aggregation and vascular smooth muscle proliferation [23, 24]. Enough NO secretion enhances vascular remodeling whereas its deficit induces attenuation in vascular remodeling [25]. It has previously been reported that conditions like atherosclerosis, hypertension, hypercholesterolemia and congestive heart failure are related to abnormal NO concentrations [26–28]. Thus, controlling NO concentration is of great importance in cardiovascular therapy and implants.

Topography has been reported to regulate the physiological behavior of cardiomyocytes. NO is a major secondary messenger in the signal transduction pathway of cardiomyocytes [29]. However, the effects of topography on NO secretion have not been elucidated. Here, were propose a novel method by which NO levels in rat cardiomyocyte cell line H9c2 are modulated in response to different size of the tantalum oxide nanodots. This is the first study which aims to study and co-relate the expressions of eNOS, iNOS in response to a nanosurface. We also studied the modulation in expression of eNOS and iNOS genes in response to the nanosurface by qPCR and the change in protein expression of Akt, eNOS, iNOS, p65 by Western Blot. We have attempted to elucidate the molecular pathways of iNOS, eNOS related cell injury as an in vitro model. We believe that understanding the response of the cell to its external environment and the upregulation or downregulation of signaling pathway in response to different size of nanodots will play an integral role in designing cardiac implants with minimal side effects and increased Bio-compatibility in the near future.

## Results

### Fabrication of nanodot arrays

**Fig. 1:**
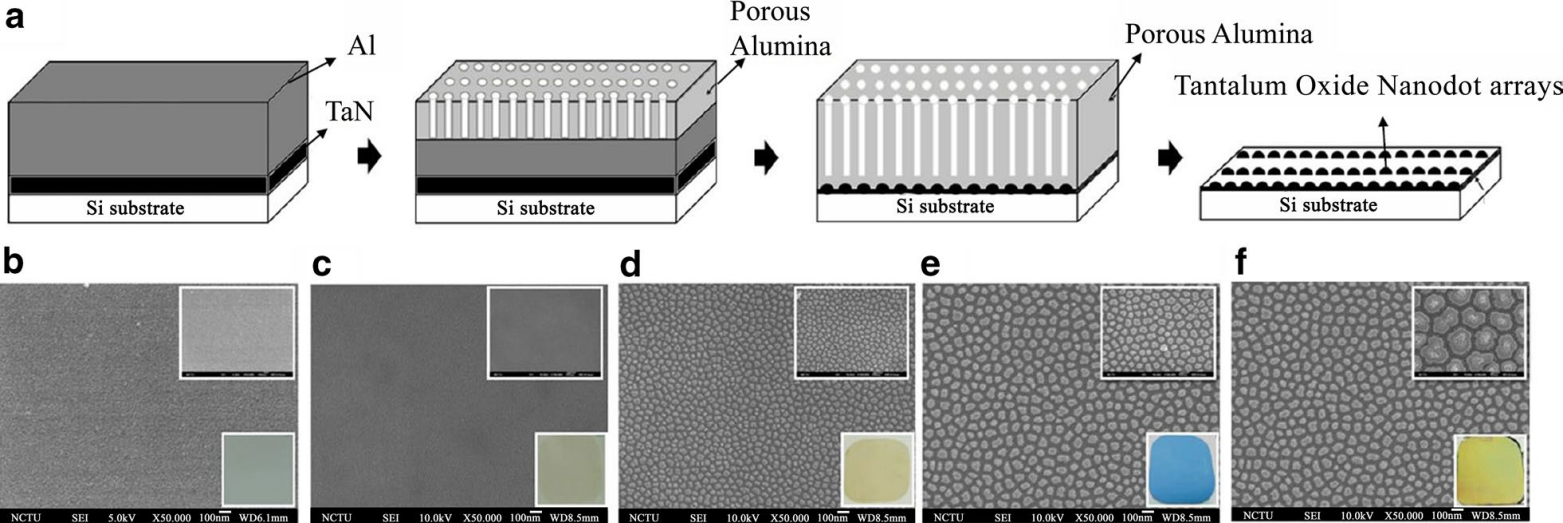
Nanodot arrays were fabricated by anodic aluminum oxide processing on Ta-coated wafer. **a** Schematic representation of the fabrication of nanodot arrays. **b** High resolution scanning electron micrographs of nanodot surface: Flat, **c** 10 nm, **d** 50 nm, **e** 100 nm, and **f** 200 nm. *Upper right* is an enlarged view and *lower right* is the appearance view. All *scale bars* 2.0 µm

Nanodots array were fabricated by anodic aluminum oxide (AAO) processing on aluminum tantalum-coated wafer (Fig. 1a). Tantalum oxide nanodots array with 10, 50, 100, and 200 nm dot diameters were constructed on silicon wafer. The nanodot diameters 12 ± 2.8, 50.35 ± 3.2, 99.4 ± 6.3, and 206.7 ± 6.5 nm were examined with scanning electron microscopy (SEM) (Fig. 1b). Dimensions of nanodots were well controlled and highly defined.

### Nitric oxide (NO) secretion exhibited a size dependent and time dependent profile

H9c2 cardiomyocytes were cultured on different nanodot arrays (Flat, 10, 50, 100, 200 nm). Aluminum coated substrate was considered as Flat. Cells treated with Lipopolysaccharide and Rapamycin served as positive and negative controls respectively. NO concentration was detected using Griess reagent system.

**Fig. 2:**
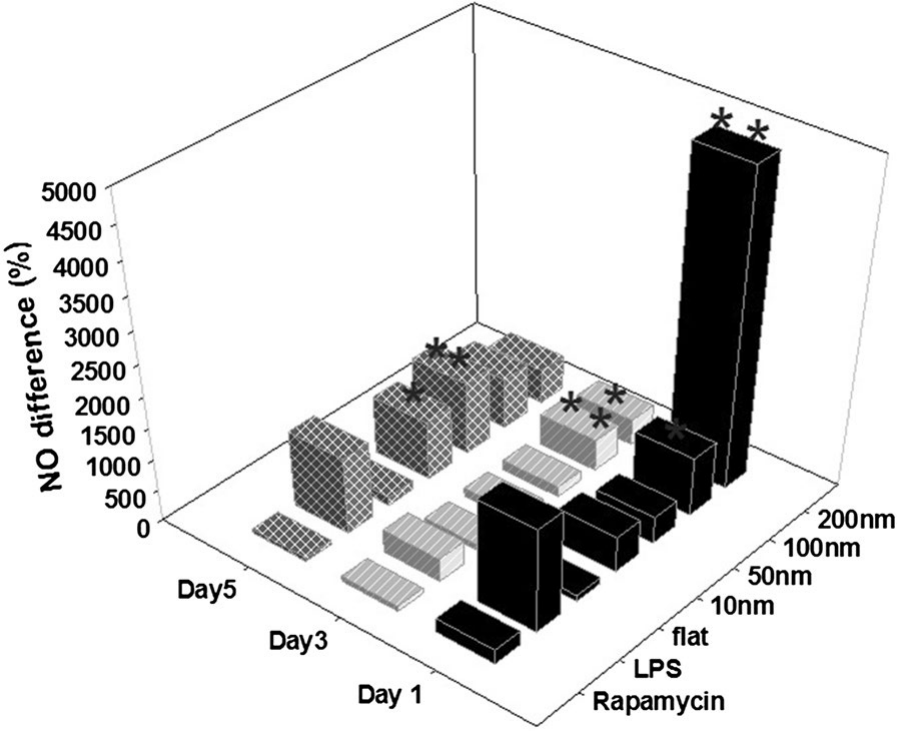
Size and time dependent profiles of NO secretion. 100 and 200 nm stimulated more NO secretion on day 1, 3. However, 10, 50 nm stimulated more NO secretion only for day 5. Lipopolysaccharide and Rapamycin are positive and negative control groups. *shows p < 0.05 and **indicates p < 0.01

NO secretion profiles displayed a size dependent relation with the nanodot arrays. After day 1 incubation period, a parabolic profile of NO secretion was observed. It increased moderately from flat to 100 nm nanodots and abruptly on 200 nm nanodot arrays. The increase on 200 nm nanodots was found out to be six folds than the control group. After day 3, the parabolic profile of NO secretion adopted a hyperbolic profile. Maximum NO secretion was displayed by cells cultured on 100 nm arrays. After day 5, hyperbolic profile was consistent. However, this time, 50 nm showed maximum NO secretion. This change was noted to be 1.5 folds when compared to the control groups (Fig. 2).

NO secretion profiles were time-dependent. After hours 24 of incubation of the cardiomyocytes, maximum stimulation of NO secretion was displayed by 200 nm nanodot arrays. After hours 72, 100 nm arrays stimulated maximum NO secretion. After hours 120, the profile remained the same but the maximum stimulation was observed to be caused by 50 nm arrays. In summary, NO secretion was stimulated by the nanodot arrays in the cardiomyocytes in a time dependent and size dependent manner (Fig. 2).

### Association of NO release and eNOS pathway with qPCR

**Fig. 3:**
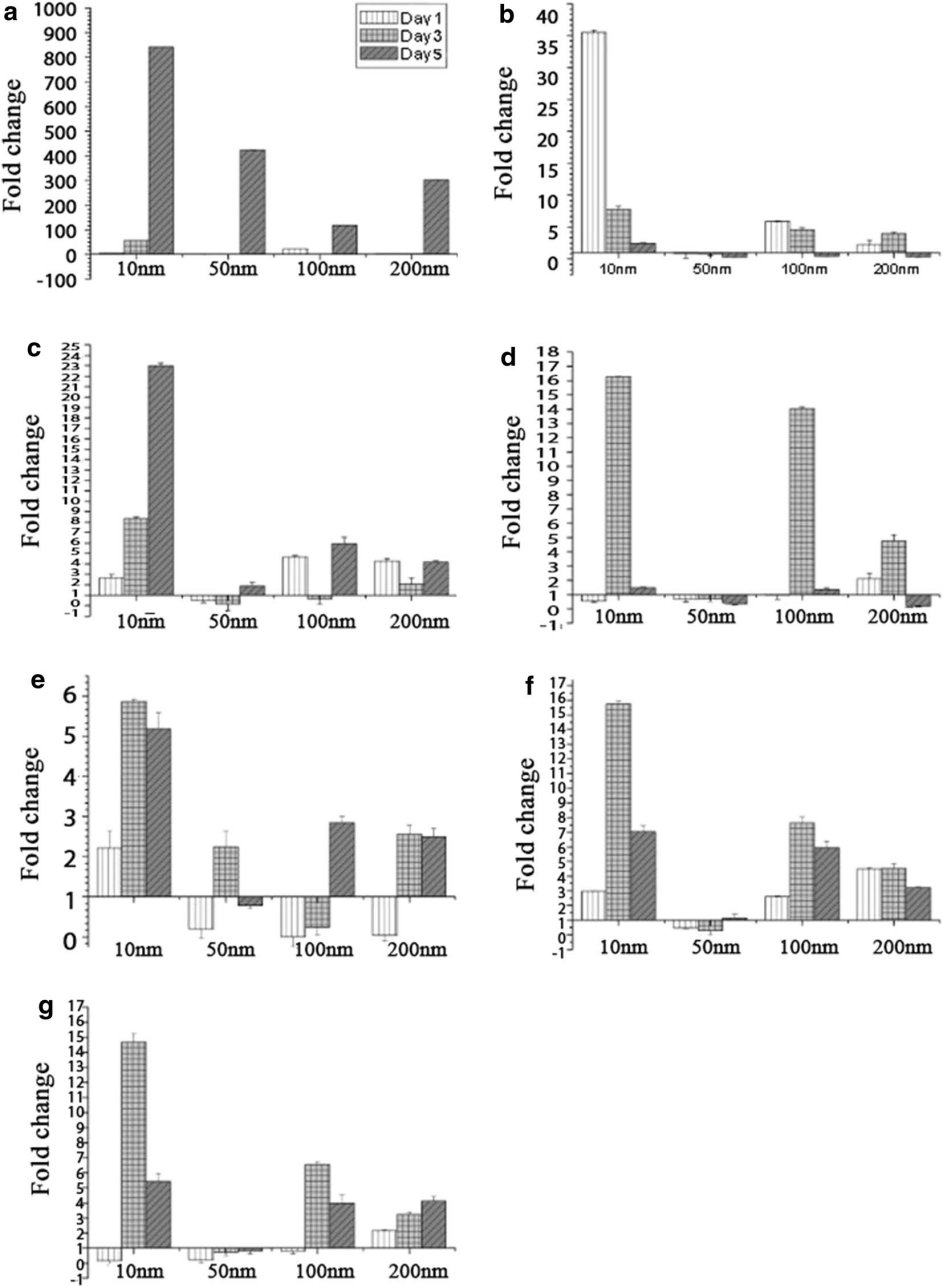
Expression of eNOS genes in H9c2 on difference nanodots arrays by qPCR. H9c2 cells were cultured on flat, 10, 50, 100, and 200 nm nanodot arrays for day 1, 3, 5 before qPCR was performed. **a** GPCR expression, **b** PI3K expression, **c** Akt expression, **d** bad expression, **e** Bcl-2, **f** NFκB (p65) expression **g** eNOS expression. The mean ± SD from at least 3 experiments is shown

The mRNA expression of eNOS pathway observed in H9c2 cardiomyocytes cultured on different nanodots was determined using qPCR (Fig. 3). According to real time PCR results, gene expression of Bad, p65 and eNOS corresponded with NO release (Fig. 3d, f, g). On day 1, Bad, p65 and eNOS were higher on 200 nm than on other surface. On day 3, those genes were higher on 10 and 100 nm; on day 5 were higher on 10 nm. The data shows apparent difference in Bad, p65. eNOS expression was correlated to NO production. In summary, with time course, the maximum amount of gene expression switched from 200 to 10 nm.

### Association of NO release and iNOS pathway

**Fig. 4:**
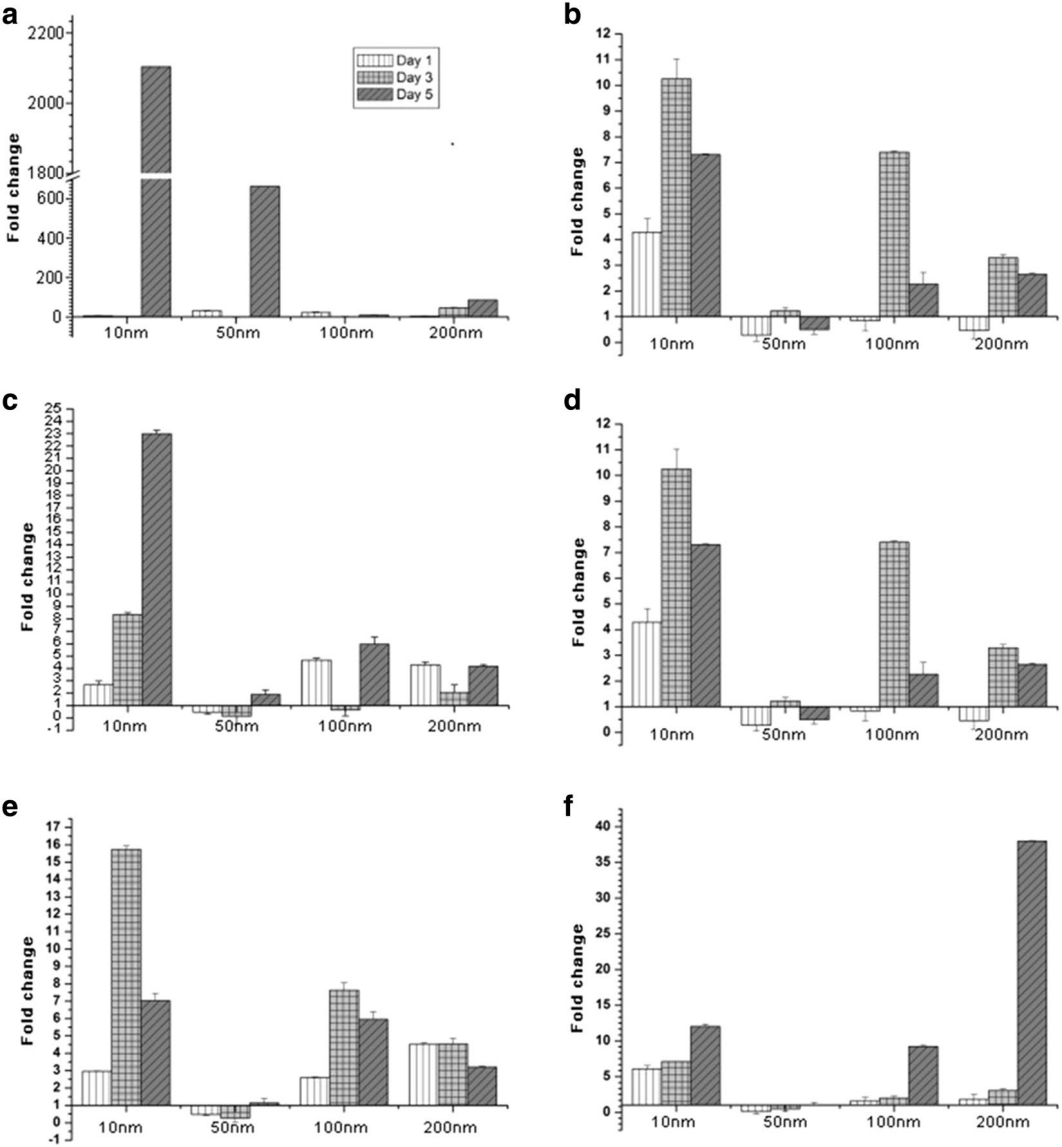
Expression of iNOS genes in H9c2 on difference nanodots arrays by qPCR. H9c2 cells were cultured on flat, 10, 50, 100, and 200 nm nanodot arrays for day 1, 3, 5 before qPCR was performed. **a** TNF-α expression, **b** ILK expression, **c** Akt expression, **d** IκBα expression, **e** NFκB (p65), **f** iNOS expression. The mean ± SD from at least 3 experiments is shown

qPCR of genes associated with inflammation related iNOS pathway (TNF-α, ILK, AKT, IκBα, iNOS) in H9c2 cardiomyocytes was performed (Fig. 4). The mRNA expression of iNOS signaling pathway genes was determined using qPCR (Fig. 4). Over various days, we observed gene expression of cardiomyocytes cultured on 50 nm nanodot arrays was significantly less than other nanodots. According to real time PCR results, gene expression of ILK, IκBα and p65 corresponded with gene regulation (Fig. 4b, d, e). However, the mRNA expression were less correlated to NO production.

### Comparison of NO secretion profile with expression patterns of genes associated with eNOS pathway by using Western Blot

The NO formation is regulated by the family of enzymes, Nitric oxide synthase (NOS) [30]. NOS have three main isoforms: neuronal NOS (nNOS), endothelial NOS (eNOS), and inducible NOS (iNOS). The cardiovascular diseases are mainly related with the eNOS and iNOS. It has previously been shown that the lipopolysaccharides and the cytokines regulate the iNOS present in the cells at the transcriptional level [31]. Some recent studies have also shown that in the iNOS pathway, NF-kB controls the iNOS expression which in-turn is targeted by Akt/ILK/TNF-α [32]. This pathway also controls the NO secretion. In the eNOS pathway, G-Protein coupled receptor (GPCR) induces secondary signal transduction pathway after receiving signals from the cytoplasm. This causes the up-regulation of Phosphoinositide 3-kinase (PI3 K) which in-turn promotes the protein Kinase B (Akt) expression. This causes the down-regulation of expression levels of pro-apoptotic protein (Bad), B cell lymphoma-2, and NF-kB. The end result is the enhanced eNOS secretion.

The survival and physiological functions of the cardiomyocytes are regulated by the phosphoinositide 3-kinase (PI3K)-Akt signaling pathway, which is one of the eNOS signal transduction pathway [33]. It has been previously shown that eNOS plays an important role in the molecular mechanisms of the development of heart disease, myocardial ischemia/reperfusion injury [34, 35].

**Fig. 5:**
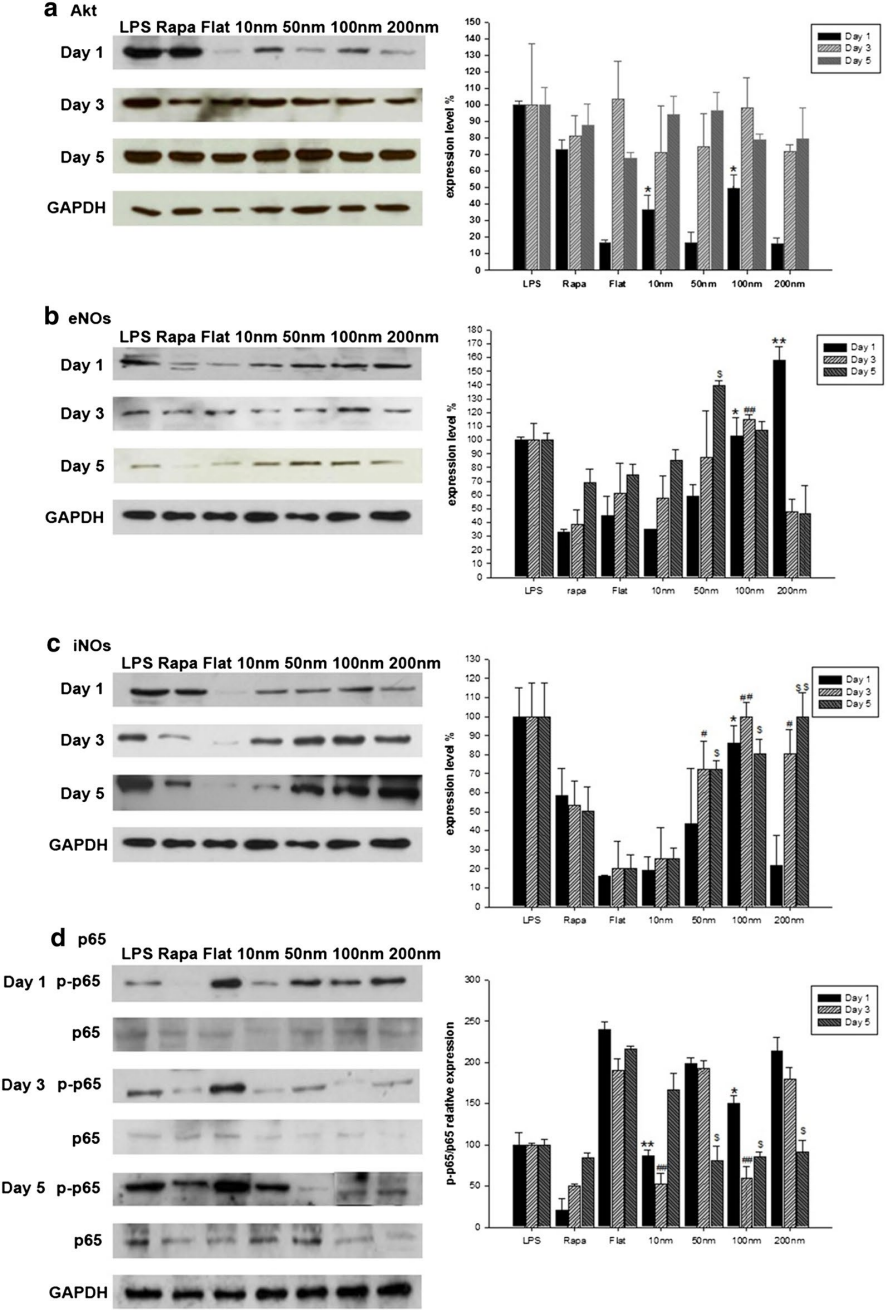
Western Blot results of NO related proteins. **a** The Akt protein expression level was upregulated on 10, 100 nm nanodot arrays after day 1 incubation. **b** The eNOS expression levels were significantly enhanced on 200, 100 and 50 nm nanodot arrays after day 1, 3, 5 incubation. **c** The iNOS expression levels were upregulated on 100 nm nanodot arrays after 1 day incubation and on 50, 100, 200 nm nanodot arrays for day 3, 5 incubation. **d** The activities of NF-kB-p65 (p-p65/p65) were downregulated on 10 and 100 nm nanodot arrays for day 1, 3 incubation period and were downregulated on 50, 100 and 200 nm nanodot arrays after incubation for day 5. *shows p < 0.05, while **shows p < 0.01. The mean ± SD from at least 3 experiments is shown

**Fig. 6:**
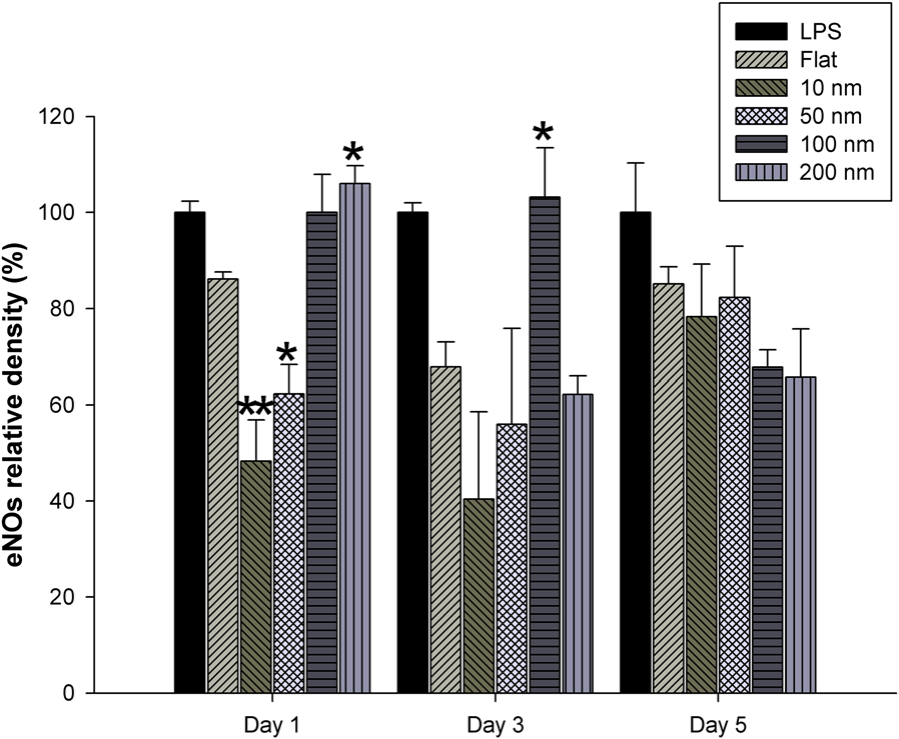
eNOS relative density over different days

Western blot of key genes associated with NO secretion (Akt, eNOS, iNOS, p65) was performed. In our findings, expression profiles of Akt and p65 showed no apparent pattern. However, the expression of Akt were globally enhanced after day 3 and day 5 incubation periods. eNOS expression profiles were highly consistent with the Nitric oxide secretion like we showed in our previous result (Fig. 5a). eNOS was highly expressed for 200 nm after incubation for day 1 whereas 100, 50 nm stimulated maximum eNOS expression after incubation for day 3 and day 5 (Figs. 5b, 6). iNOS expression profiles did not show much resemblance with the NO secretion (Fig. 5c). Therefore, it is quite likely that the NO secretion of the cells grown on the nanodot arrays was dominated by the eNOS signaling pathway than by the iNOS pathway.

Furthermore, we checked the expression of p65 for day 1, 3 and 5. The activity of p65 was calculated by dividing the expression of p-p65 with the expression level of p65. The calculated p65 activity was roughly consistent with the NO secretion. It showed high activity for 200 nm for day 1 and later showed a size dependent expression (Fig. 5d).

## Discussions

Nitric oxide is a unique signaling messenger in cardiomyocytes [36]. Various research groups have studied its vasodilation effects [37] and regulation of the platelet aggregation in the past [38]. Meanwhile, NO molecules can also promote the expression of pro-angiogenic cytokines, favorable for angiogenesis [39]. Therefore, modulation in level of NO by cardiomyocytes in response to a vascular implant is of great interest to the engineers. Nanosurfaces have been seen to stimulate and modulate the NO secretion [40]. However, a size and time based study of the modulation in NO secretion in response to different sized nanosurfaces has not been done before. Here, we provided the evidence for the first time that nanodot arrays of different sizes can stimulate the NO release to a different extent in rat cardiomyocytes cell line H9c2 (Fig. 2). Our results from day 1 showed that the cardiomyocytes display a size dependent production of NO with 200 nm arrays showing the maximum production and 50 nm, the least. This proves that the cells respond differently to different nanodot arrays by secreting different amounts of NO (Fig. 2). To study the modulation in NO secretion by the cells in response to different nanodot arrays over time, we measured the NO secretion profile over 5 days. Our observations from day 3 and day 5 showed that the cardiomyocytes not only have a size-dependence with the nanodot array but also a time dependent relation (Fig. 2). Variation in cell characteristics in response to different surface roughness and geometry has been shown before [41–43]. In the present study, we measured the cardiomyocyte response to the nanodot arrays of size ranging from 10 to 200 nm. We attempted to fabricate 5 nm nanodot arrays as well. However, their average size was fairly close to 10 nm (7 nm) and therefore, we decided to exclude it from our study. In our previous studies, we have already studied the modulation of characteristics of H9c2 in response to nanodot arrays [3]. Our findings in the past showed that with the increase in the size of the nanodots, the cell density decreases along with apoptosis like appearance followed by decreased number of focal adhesions which individually as well as together, are indicator of unhealthy cells. Hence, nanodot arrays from 10 to 200 nm were chosen as the size limits for the experiments in this study.

Secondly, to elucidate the signaling pathway responsible for the regulation and shifting of NO secretion over the subsequent days, we performed q-PCR of GPCR, PI3 K, Akt, Bad, Bcl-2, NF-KB, responsible for the eNOS expression (Fig. 3) and of TNF-α, ILK, Akt, IκBα, NF-KB responsible for the iNOS expression (Fig. 4). BCL-2 is an anti-apoptotic member of family BCL-X. Activated BAD induces apoptosis by inhibiting BCL-2 [44] and BCL-2 has been seen to downregulate Transcription factor NF-kB [45]. eNOS triggers the expression of NO [46]. It has been seen that activated Pi3K/Akt signaling pathway can impair NF-kB signaling [47]. Our findings showed that the expression of genes triggering eNOS (Fig. 3) was highly consistent with the maximum NO secretion for all the days but the genes triggering iNOS were not found to be consistent with the NO secretion (Fig. 4). This made us reach the conclusion that for these nanodot arrays, the NO secretion is modulated by the eNOS signaling pathway. NO triggered by eNOS plays a vital role in regulating vasodilation, anti-thrombic actions, apoptosis [48]. Previous studies have shown that eNOS deficiency can cause cardiomyocytes apoptosis which can be a factor in causing congenital heart defects during development [49]. To further validate that the NO modulation in the cells was triggered by eNOS, we performed Western Blot of the protein expression of eNOS, iNOS and the genes triggering them (Fig. 5). The protein expression of eNOS was found to be consistent with NO secretion profile for all the time periods. However, iNOS was found to be inconsistent.

**Fig. 7:**
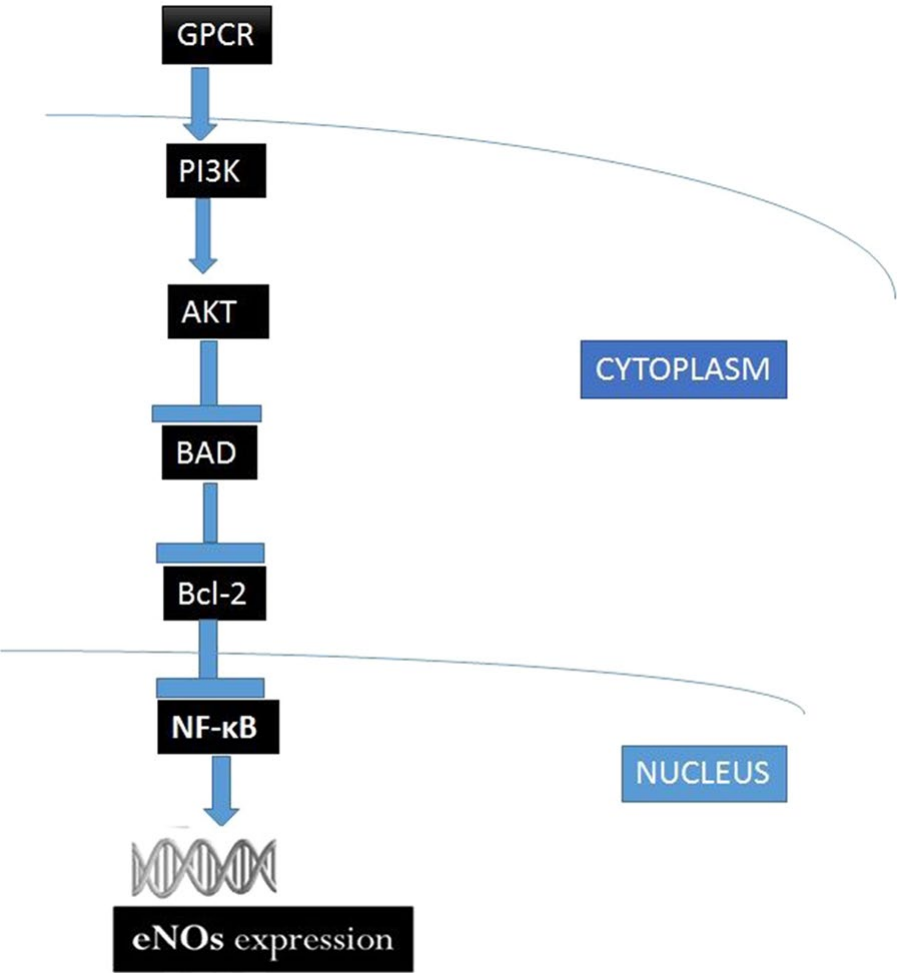
Possible gene regulation for NO modulation by eNOS upregulation

Surprisingly, we observed changes in the expression of iNOS as well as eNOS. We found that for day 1, eNOS modulates the NO expression in the cardiomyocytes. However, for the subsequent days, NO has a negative regulatory effect on the eNOS and iNOS expression as shown in the previous studies [50–53]. As one may notice, the highest eNOS expression on day 1 results in the highest NO secretion (Fig. 2). However, the high concentration of NO causes the decreased eNOS expression on day 3 for 200 nm. Similar results were obtained on comparing the NO secretion profile (Fig. 2) and eNOS protein expression (Fig. 5b) for subsequent days. This showed that NO as well as eNOS play a very crucial role in regulating each other. Similar observations can be made from the western blot expression of iNOS and NO secretion. NO has a negative regulatory effect on the iNOS expression (Fig. 5c). On day 1, maximum NO secretion was observed for 200 nm nanodot arrays (Fig. 2). This causes the decreased expression of iNOS on day 1 Similar results were obtained on comparing the NO secretion profile (Fig. 2) and iNOS protein expression (Fig. 5c) for subsequent days. Even though our data suggests that nanodot arrays can alter the eNOS and iNOS genes, nevertheless, more experiments need to be done to elucidate their relative upregulation/downregulation (Fig. 7).

Due to their strength, flexibility and based on their biocompatibility, nanomaterials are continuously exploited to make vascular stents. Stents serve the purpose of keeping a blood vessel open and are therefore employed to treat Coronary heart disease like Atherosclerosis. In the past decade, properties of multiple materials have been studied for application in the field of Biomedical engineering. Out of these, Titanium (Ti), stainless steel, alloy of Cobalt-Chromium have been extensively studied due to their mechanical and biocompatible properties [54, 55]. Alloy of Cobalt-Chromium-Molybdenum provide better wear resistance [56]. Use of Tantalum (Ta) as an implant material has also been studied in the past due to its excellent X-Ray visibility and low magnetic susceptibility due to which it is often used as X-ray markers for stents [57]. Nevertheless, stents have been observed to undergo narrowing due to VSMC proliferation in response to injury at the time of stent implantation [58, 59]. Use of Ti as a sent has been widely studied by many researchers in the past. In 2008 Karla et al. studied the interactions between Bovine Aortic endothelial cells and Titanium dioxide (TiO_2_) nanotubes along with flat Ti surfaces and observed better focal adhesions, high ECM, unidirectional actin and enhanced lamellipodia formation along with higher NO secretion in contrast to flat Ti surfaces which proves the ability of the nanotopography to control the cell characteristics and NO secretion [40]. A similar study was performed by Margherita et al. [60] to elucidate the effect of TiO_2_ nanotopography in the differentiation process of PC12 cells and the involvement of NOS and eERK in the same.

In summary, the present study exploited the use of Tantalum oxide nanodot arrays (Fig. 1) as a potential material for making cardiac stents by measuring the NO release in response to the nanodots as a measure of size and time (Fig. 2). Modulation in the expression of key genes regulating the NO release was also elucidated (Figs. 3, 4). Western Blot was performed to study the protein expression of key genes responsible for the modulation of NO (Fig. 5). The modulation was attributed to eNOS (Fig. 6). We also observed the negative regulatory effect of NO on eNOS and iNOS (Fig. 5) which is consistent with the previous studies.

These results prove an effective way to modulate the NO expression in cardiomyocytes on coming in contact with a cardiac implant. The modulation of NO by the implant will have a high degree of impact on the health of the heart cells of the patients. Moreover, the activation of eNOS has also been shown in previous studies to provide atheroprotection [61]. Hence, these findings will be of great importance in designing the surface of the implants within the fields of biomaterial and tissue engineering.

## Conclusions

In the present study we have shown that the nano-topography modulates the NO secretion levels in a time as well as size dependent manner in the rat heart cell line H9c2. The stimulation of NO secretion by the nanodot arrays was found to shift from 200 nm on day 1 to 50 nm on day 5 (Fig. 2). We also attempted to identify the genes responsible for the NO secretion in response to our nanodot arrays with the help of q-PCR. Our findings show that the expressions of Bad, p65, eNOS were consistent with the NO secretion over different days. The eNOS protein expression was consistent with the NO secretion profile over different days supporting the q-PCR data of the eNOS genes (Fig. 5). The iNOS expression level was not found to be consistent with the NO secretion. This led us to conclude that the NO secretion in the cardiomyocytes cultured over the nanodot arrays is primarily regulated by the eNOS signaling pathway rather than the iNOS pathway. In addition, NO was also found to have a negative regulatory effect on the eNOS as well as iNOS expression.

According to the results in this study, the 50, 100 nm nanodot arrays can be the suitable nanotopography pattern for heart stent design. These two nanodot surfaces promote NO secretion which can increase the vascular smooth muscle relaxation at the same time. The results of this study can improve the heart stent design in the medical treatments.

## Methods

### Chemicals

Lipopolysaccharide (LPS), Rapamycin, Trypsin were purchased from Sigma-Aldrich (USA). Bovine serum albumin (BSA) was purchased from GIBCO (Thermo Fisher Scientific Inc. USA). Phosphate buffered saline (PBS) was purchased from Bio-tech (Taipei, Taiwan). Sulfuric acid (H_2_SO_4_), oxalic acid (H_2_C_2_O_4_), and phosphoric acid (H_3_PO_4_) were purchased from Sigma Chemicals (Perth, Western Australia), 6-inch silicon wafers, Aluminum ingots were purchased from Admat-Midas (Norristown, PA, USA). Other chemicals of analytical grade or higher were purchased from Sigma or Merck (USA).

### Fabrication of nanodot arrays

Nanodot arrays were fabricated as previously described [3]. A 200-nm-thick tantalum nitride (TaN) thin film was sputtered onto a 6-in silicon wafer (Summit-Tech, West Hartford, CT, USA) followed by deposition of 3 µM-thick aluminum onto the top of a TaN layer by thermal coater. Highly uniform nanodot arrays were fabricated from 10 to 200 nm. Nanodot arrays of size smaller than 10 nm could not be fabricated due to technical limitation. Anodization was carried out in 1.8 M sulfuric acid at 5 V, 90 min for the 10 nm nanodot array and in 0.3 M oxalic acid at 25 V, 90 min for the 50 nm. 100 and 200 nm nanodot arrays were fabricated by a two-step anodization method. In the first anodic oxidation step, anodization was carried out in 0.3 M oxalic acid at 40 Volts, 10 min, for 100 nm nanodot array and in 5 % (w/v) H_3_PO_4_ at 100 V, 5 min, for 200 nm nanodot arrays. The porous alumina was removed by immersion in 5 % (w/v) H_3_PO_4_ for 70 and 60 min for 100 and 200 nm nanodot arrays, respectively. Second anodization step is repeated in the same way. Porous anodic alumina was formed during the anodic oxidation. The porous alumina was removed by immersing in 5 % (w/v) H_3_PO_4_ overnight. The dimensions and homogeneity of nanodot arrays were measured and calculated from images taken by JEOL JSM-6500 TFE-SEM. A thin layer of platinum was sputtered onto the structure.

### Cell culture

To eliminate possible contamination of nano/micro particles, H9c2 cells were cultured in Dulbecco’s Modified Eagle’s Medium complimented with 10 % FBS and 5 % CO_2_ and incubated at 37 °C. The cell culturing was performed in a class-10 clean room.

### Nitrite (NO) assay (Griess reagent system)

Griess reagent system was purchased from Promega. Cells (5 × 10^3^ cells/cm^2^) were seeded on different nanodot arrays (flat, 10, 50, 100, 200 nm). Production of NO was assayed by measuring the stable metabolite of nitrite levels in the culture medium. Dispense 50 µL of the 1 % Sulfanilamide Solution to all experimental samples and incubate for 5–10 min at room temperature, protected from light. Then dispense 50 µL of the NED Solution (0.1 % naphthylethylenediamine dihydrochloride/2 % H_3_PO_4_) and incubate at 25 °C for 10 min. The absorbance at 550 nm was measured with UV-spectrophotometer.

### Quantitative real-time PCR

The quantitative Real time PCR was performed to investigate the nanotopographic effects on the gene expression level. Oligo primers of the genes (TNF-a, ILK, AKT, IκBα, p65, iNOS, GPCR, PI3 K, Bad, Bcl-2, eNOS) are listed in the Table 1 [46, 47, 62–66]. 0.5 µg/µL LPS with hours 3 pre-treatment was used as a positive control. 100 nM Rapamycin for hours 2 was used as a negative control group. The specificity of primers was verified with polymerase chain reaction with H9c2 reverse transcribed mRNA as a template.

**Table 1:** Primer sequence used for RT-PCR

Gene name	Forward	Reverse
TNF-α	TCTGTCTACTGAACTTCGGGGTGAT	CAGCCTTGTCCCTTGAAGAGAACC
GPCR	GCGCGGATCCGCCACGATGCTTGTCCTGCG	GCGCGAATTCTTAGGAGCTTAGTCTACAAACTG
ILK	GCTCAACTTTCTGGCAAAGC	TGTGGCAAGTGACAAAGCTC
PI3K	TGACGCTTTCAAACGCTATC	CAGAGAGTACTCTTGCATT
Akt	GTGCTGGAGGACAACGACT	GTGTAGGGTCCTTCTTGAGCA
IκBα	GACGAGGATTACGAGCAGAT	CCTGGTAGGTTACTCTGTTG
Bad	CCCCCCAATCTCTGGGCAGCG	TCACTGGGAGGGATGGA
Bcl-2	GCTACGAGTGGGATACTGG	GTGTGCAGATGCCGGTTCA
p65	CATTGAGGTGTATTTCACGG	GAACACAATGGCCACTTGCC
eNOs	TGGCAGCCCTAAGACCTATG	AGTCCGAAAATGTCCTCGTG
iNOs	CTGCATGGAACAGTATAAGGCAAAC	AGACAGTTTCTGGTCGATGTCATGA

All sequences are from 5′ to 3′

Total RNA was extracted from 5 × 10^3^ cells using TRI-reagent (Talron Biotech) according to the manufacturer’s specifications. The RNA was isolated using 200 μL chloroform extraction and isopropanol precipitation. The RNA extract was immediately purified using an RNeasy Mini Kit (Qiagen) to remove impurities and unwanted organic compounds. Purified RNA was re-suspended in DEPC-treated water and quantified by OD260. The OD260-to-OD280 ratio usually exceeded 2.0 at this stage. For cDNA synthesis, 29 μL total RNA was annealed using 1 μg oligo-dT primer, followed by reverse transcription using SuperScript-III Reverse Transcriptase (Invitrogen) in a total volume of 50 μL. Between 0.2 and 0.5 μL of the reverse transcription reactions were used for quantitative qPCR using SYBR Green I performed on an iCycler iQ5 (Bio-Rad Laboratories). The cycling conditions were as follows: 1 cycle of 5 min at 95 °C and 50 cycles of 20 s at 95 °C, 20 s at 55 °C, and 40 s at 72 °C. Fluorescence was measured after each 72 °C step. Expression levels were obtained using threshold cycles (Ct) that were determined by the iCycler iQ Detection System software. Relative transcript quantities were calculated using the ΔΔCt method. The gene GAPDH was used as a reference gene and was amplified along with the target genes from the same cDNA samples. The difference in threshold cycles of the sample mRNA relative to GAPDH mRNA was defined as the ΔCt. The difference between the ΔCt of the control flat and the ΔCt of the cells grown on nanodot arrays was de fined as the ΔΔCt. The fold change in mRNA expression was expressed as 2ΔΔCt. The results were expressed as the mean SD of six experiments.

### Protein extraction and Western blot analysis

Total protein was extracted from 5 × 10^3^ cells using lysis buffer. Ultrasound was applied using a sonicator operating the sample 6 min at 4 °C. Cultured H9c2 cardiomyocytes were lysed and centrifuged at 3000 rpm for 2 min. The supernatants were transferred to new Eppendorf tubes and protein concentrations were defined using UV/VIS spectroscopy (595 nm). After the protein concentrations were defined, solutions were mixed with 5X sample buffer and lysis buffer to a final concentration of 1 mg/ml. Samples were heated at 95 °C for 3 min and cooled at 4 °C for 3 min, which was repeated three times. Proteins were separated using 10 % SDS-PAGE gels and transferred to PVDF membranes. Non-specific protein binding was blocked by a 5 % milk solution for hours 2. The membranes were subsequently blotted at 4 °C overnight with the following primary monoclonal antibodies: (1) anti-eNOS (1:1000; Abcam); (2) anti-iNOS (1:500; Abcam); (3) anti-p65 (1:250; Genetex); (4) Anti-phospho-NFκB p65(Ser536) (1:1000; cell signaling); (5) anti-Akt;(6) GPPDH (1:200; Abcam), which were diluted in blocking buffer. Specific primary anti-bodies were blotted using second antibodies (1:10,000) in the blocking buffer at room temperature for hours 2. Chemiluminescent detection was performed using western blotting luminol reagent and oxidizing reagent (USA). To compare the different groups, densitometric quantification was performed only on equally processed blots. Bands on Western blots were analyzed with a Scan Jet 3390 computing densitometer using IMAGE J software. Relative densities of immunoreactive bands were normalized to the density of corresponding bands for GAPDH. The results were expressed as the mean SD of three experiments.

### Statistics

The results are expressed as the mean ± SD of three experiments. Student’s t test was employed to determine data sets that differed significantly from one another, and significance was defined as a p value <0.05; highly significance was defined as a p value <0.01.

## Abbreviations

AAO: anodic aluminum oxide
cDNA: complementary DNA
eNOS: endothelial nitric oxide synthase
iNOS: inducible nitric oxide synthase
LPS: lipopolysaccharide
nm: nanometer
nNOS: neuronal nitric oxide synthase
SD: standard deviation
NO: nitric oxide
OD: optical density
PBS: phosphate buffered saline
PI3K: phosphoinositide 3-kinase
PVDF: polyvinylidene fluoride
q-PCR: quantitative polymerase chain reaction
Rpm: revolutions per minute
RT-PCR: reverse transcriptase polymerase chain reaction
TaN: tantalum nitride
